# London Fogs

**Published:** 1901-11-02

**Authors:** 


### London Fogs.

The County Council have agreed to contribute £250 towards an inquiry by the Meteorological Council into the occurrence and distribution of fogs in London. This is undoubtedly a step in the right direction, from a scientific point of view, and it is just possible that in the future the advent of fogs will be foretold with about as much accuracy as other weather forecasts. The main practical fact is that a London fog differs from one in the country or on the sea simply because it entangles in its meshes smoke. It so happens that the season for fogs all over the country coincides with the renewal of fires after the autumn, and thus it arises that on many days in the last three months of the year the atmosphere of London is of the colour and consistency of pea-soup. The colour and the pungency of London fogs are caused by the carbonaceous, sulphurous and other products of the imperfect combustion of coal which are condensed in and upon the aqueous globules on which the opacity of all fogs depends. The loss to the community, due to the hindrance to the traffic, is very great, and from a medical point of view it is difficult to conceive a more unpleasant or irritating mixture to inhale. The practical results are congestion and inflammation along a part, or the whole, of the respiratory tract. Hence the foggy season is the time for colds in the head, sore throats, and bronchitis, while asthmatics may or may not find their usual condition accentuated according to their individual idiosyncrasies. From a theoretical point of view it is a little difficult to apportion the ill-effects to the various constituents of the fog, but while the damp acts in a similar manner in London as elsewhere the smoke irritates the respiratory tract, the eyes, and indeed every part to which it gains access, and therefore renders them more vulnerable to the attacks of disease. It is obvious that the damp half of the fog cannot be prevented, even if it can be successfully forecast, so we can confine our attention to the smoke element.

The difficulty lies in the fact that the bulk of the smoke is produced by domestic fireplaces and not by great factories which, to a certain extent, can be controlled, and at present no satisfactory grate has been designed capable of maintaining a cheerful open fire and at the same time of consuming its own smoke. A mixture of coal and coke produces greater heat and less smoke, but it requires more attention and care than as a rule is given to fires, except those in rooms constantly occupied. Peat is a very dirty form of fuel, and so is not suitable for domestic use on a large scale. Electric fires are out of the question on account of their excessive cost, and so we are reduced to gas, which has many advantages but is not cheerful. Gas and air mixed form a very hot flame, and when this is burnt beneath pieces of asbestos in an ordinary fireplace the result is a very convenient, cleanly, and easily-regulated form of fire. No doubt the universal substitution of this for ordinary coal would practically abolish fogs altogether; but this cannot be hoped for since a gas fire has not a cheerful appearance in a sitting-room, although it is far more convenient than coal in a bedroom. Again, cooking can be as easily and successfully carried on by gas as regards baking and boiling, but roasting by a ring of naked gas jets does not seem to us to give such a pleasant taste to the meat as when an open fire is used.

The question of relative expense rests upon the cost of gas and the care of the servants. It has been more than once suggested that if gas were produced at the pit mouth and brought to London in pipes the initial outlay for mains would be balanced in a very short time by the decreased cost of the gas. It is also claimed by the latest exponent of this scheme that the recovery of the bye-products from coal—tar and coke—would become a more profitable industry than at present and that the latter could be carried to London at a far less cost and used in connection with gas to form fires which would ensure complete combustion without smoke. The economy in the cost would lead to the adoption of gas wherever possible in preference to coal and so to the abolition of fogs. Whether we eventually carry our gas for miles across country, as several of the large towns do their water, or not, there can be no doubt that it would be a great benefit to many densely-populated neighbourhoods if the gasworks were removed from them and the air thus freed from sulphuretted hydrogen and other unpleasant products of gas-making.

